# Physiological responses to acute cold exposure in young lean men

**DOI:** 10.1371/journal.pone.0196543

**Published:** 2018-05-07

**Authors:** Francisco M. Acosta, Borja Martinez-Tellez, Guillermo Sanchez-Delgado, Juan M. A. Alcantara, Pedro Acosta-Manzano, Antonio J. Morales-Artacho, Jonatan R. Ruiz

**Affiliations:** 1 PROFITH “PROmoting FITness and Health through physical activity” research group, Department of Physical Education and Sport, Faculty of Sport Sciences, University of Granada, Granada, Spain; 2 Department of Medicine, Division of Endocrinology, Einthoven Laboratory for Experimental Vascular Medicine, Leiden University Medical Center, Leiden, the Netherlands; 3 Department of Physical Education and Sport, Faculty of Sport Sciences, University of Granada, Granada, Spain; Institute of Zoology, CHINA

## Abstract

The aim of this study was to comprehensively describe the physiological responses to an acute bout of mild cold in young lean men (n = 11, age: 23 ± 2 years, body mass index: 23.1 ± 1.2 kg/m^2^) to better understand the underlying mechanisms of non-shivering thermogenesis and how it is regulated. Resting energy expenditure, substrate metabolism, skin temperature, thermal comfort perception, superficial muscle activity, hemodynamics of the forearm and abdominal regions, and heart rate variability were measured under warm conditions (22.7 ± 0.2°C) and during an individualized cooling protocol (air-conditioning and water cooling vest) in a cold room (19.4 ± 0.1°C). The temperature of the cooling vest started at 16.6°C and decreased ~ 1.4°C every 10 minutes until participants shivered (93.5 ± 26.3 min). All measurements were analysed across 4 periods: warm period, at 31% and at 64% of individual´s cold exposure time until shivering occurred, and at the shivering threshold. Energy expenditure increased from warm period to 31% of cold exposure by 16.7% (P = 0.078) and to the shivering threshold by 31.7% (P = 0.023). Fat oxidation increased by 72.6% from warm period to 31% of cold exposure (P = 0.004), whereas no changes occurred in carbohydrates oxidation. As shivering came closer, the skin temperature and thermal comfort perception decreased (all P<0.05), except in the supraclavicular skin temperature, which did not change (P>0.05). Furthermore, the superficial muscle activation increased at the shivering threshold. It is noteworthy that the largest physiological changes occurred during the first 30 minutes of cold exposure, when the participants felt less discomfort.

## Introduction

When humans are exposed to cold, they exhibit mainly two types of physiological responses in order to protect their core temperature. They can i) rely on their body insulative properties by changes in blood perfusion to decrease heat loss; and/or ii) increase their energy expenditure up to 3–5 fold above the resting energy expenditure (REE) [1], in order to counterbalance heat lost (cold induced thermogenesis, CIT) [2].

Human CIT response has been studied broadly using different methods (cold air, water immersions, water perfused garments, ice-blocks, among others), as well as different exposure times [1]. CIT is typically divided into 2 main components: shivering thermogenesis and non-shivering thermogenesis. Shivering thermogenesis increases heat production in response to cold through muscular contractions. It is able to provoke a 5-fold increase in REE in humans, being fundamental to avoid hypothermia and ensure human survival under extreme cold conditions [3–5]. However, shivering thermogenesis is often uncomfortable and fatiguing, and compromises locomotion. On the other hand, non-shivering thermogenesis is triggered mainly by mild cold and can increase REE to an extent of ≈ 30%, without inducing a large discomfort [6]. Consequently, it has become an attractive target as a health-promoting stimulus to counteract obesity and related comorbidities [7–10].

Through many decades, researchers have focused mainly on characterizing the metabolic effects of severe cold exposure, especially in shivering thermogenesis [11]. Nevertheless, it remains unclear how non-shivering thermogenesis is regulated and which are the mechanisms involved during mild cold exposure. Important gaps, such as metabolic pathways and fuel selection need to be further examined. Moreover, the relative contribution of different tissues to non-shivering thermogenesis is still to be discerned. Brown adipose tissue (BAT), a thermogenic tissue with the ability to oxidize carbohydrates and lipids and to dissipate energy in the form of heat, seems to play a key role in non-shivering thermogenesis [12–14]. Estimations suggest that cold-activated BAT could account itself for 2.5–5% of the increase in REE [2]. However, there is an important fraction of body heat production that is missing. Recent findings suggest that other tissues such as the skeletal muscle may also play a key role in non-shivering thermogenesis [7,15], yet more evidence is needed. Moreover, even less is known about the role of subcutaneous white adipose tissue over non-shivering thermogenesis.

For a better understanding of the underlying mechanisms of non-shivering thermogenesis, a comprehensive analysis of the cold induced physiological responses is required. Consequently, in the present study we described the physiological responses to an acute bout of mild cold exposure until shivering occurred in young lean men. Specifically, we analysed the changes in energy expenditure and substrate metabolism, skin temperature and thermal comfort perception, superficial muscle activity, hemodynamics of the forearm and abdominal regions, and heart rate variability. The changes on these variables were analysed at several temperature time points during an individualized cooling protocol, which encompassed the whole spectrum of non-shivering thermogenesis.

## Material and methods

### Study participants

A total of eleven Caucasian male adults (age: 23 ± 2 years; body mass index: 23.1 ± 1.2 kg/m^2^; lean mass index: 17.1 ± 1.2 kg/m^2^; fat mass index: 4.5 ± 0.9 kg/m^2^) participated in this experimental trial (ClinicalTrials.gov, ID: NCT02365129). All participants were healthy, non-smokers, and did not take any medication that could have altered their energetic or neuromuscular responses to cold exposure. The study protocol and the informed consent were performed in accordance with the Declaration of Helsinki (revision of 2013). The study was approved by the Human Research Ethics Committee of the University of Granada (n° 924) and of the “Servicio Andaluz de Salud” (Centro de Granada, CEI-Granada). The study was conducted between March and April 2016.

### Procedures

**Fig 1:**
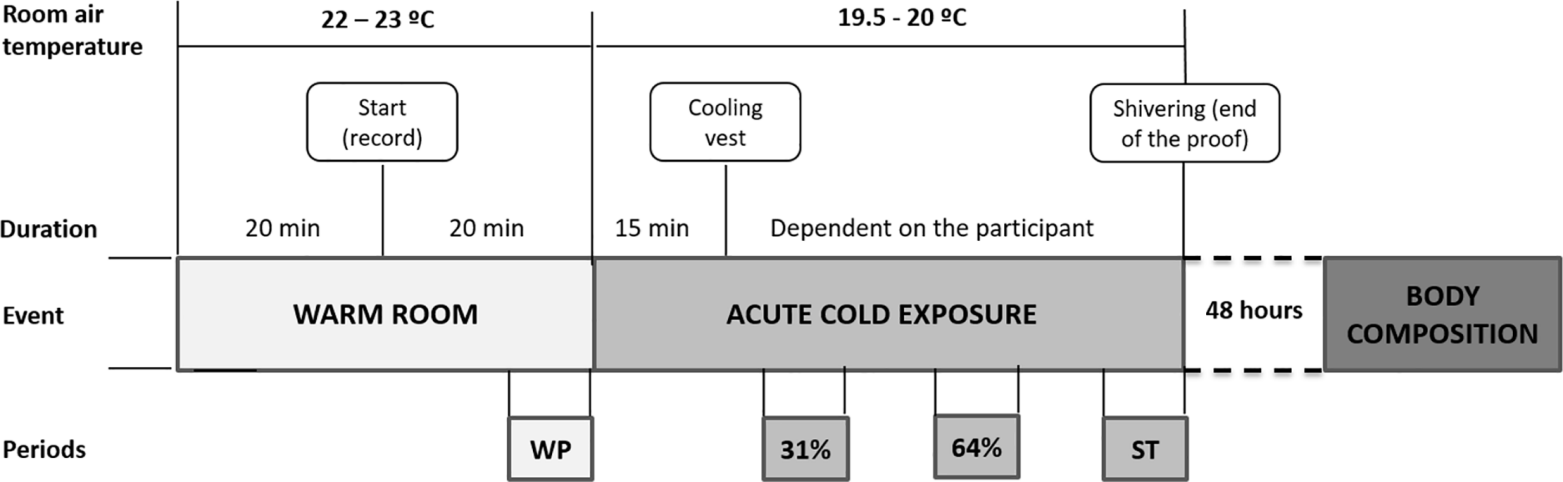
Study protocol.

The study protocol is shown in Fig 1. The participants were advised (i) not to change their sleeping habits, (ii) to refrain from any moderate (within the previous 24 hours) or vigorous physical activity (within the previous 48 hours), and (iii) not to drink alcoholic or stimulant beverages (within the previous 6 hours). In addition, they were advised to arrive at the centre in fasting conditions (at least 8 hours). The assessments took place between 8.30h - 16.00h, except in the case of two participants who were assessed between 16.30h - 20.00h.

At their arrival, the participants confirmed that they followed all the pre-study conditions. They voided their bladders and dressed in standardized clothes (sandals, shorts, and T-shirt, clo-value: 0.20). Weight and height were measured (Seca, Hamburg, Deutschland). The participants entered a warm room (22.7 ± 0.2°C) where they lay on a reclined bed for 40 minutes. They were not allowed to stand up, move or rub their bodies, or to fall asleep. Afterwards, they entered an air-conditioned room (19.4 ± 0.1°C) and lay on a bed in the same position. Fifteen minutes after entering the cold room, the participants put on a temperature controlled water perfused cooling vest (Polar Products Inc., Ohio, USA), which covered the clavicular region, as well as their chest, abdominals, and back. Water temperature started at 16.6°C, and decreased ~ 1.4°C every 10 minutes until shivering occurred. The shivering threshold was determined visually and by asking the participants if they were experiencing shivering. The cold exposure protocol was recorded on video and trained researchers further confirmed shivering onset.

The participants lay quietly in the warm room to acclimate to the environmental temperature (22–23°C) for 20 minutes. Then, from minute 21 to 40, gases exchange parameters, skin temperature, hemodynamics of the forearm and abdominal regions, and heart rate variability were recorded. When the participants were moved into the cold room, the same measurements were made from minute one until shivering occurred. Electromyography was recorded between minutes 31–40 in the warm room, and during the whole cold exposure. We also assessed the thermal comfort perception in both warm and cold conditions.

### Physiological measurements

#### Resting energy expenditure and substrate metabolism

Gases exchange parameters were recorded by indirect calorimetry using a breath-by-breath technique metabolic cart (CCM Express, Medgraphics Corp, St Paul, Minneapolis, USA). A neoprene face-mask equipped with a directconnect™ metabolic low flow sensor (Medgraphics Corp, Minnesota, USA) was used for gases collection. Flow calibration was performed using a 3 L calibration syringe at the beginning of every testing day. Furthermore, the metabolic cart was re-calibrated every 30 minutes using two standard gas concentrations following the manufacturers´ instructions.

Raw data were obtained every 10 seconds using the Breeze Suite software (Medgraphics Corp, St Paul, Minneapolis, USA). REE and respiratory quotient were calculated from the readouts of oxygen consumption volume and carbon dioxide production volume. REE was calculated according to the equation proposed by Weir [16], and substrate metabolism was estimated following the equations reported by Frayn [17] and Schadewaldt et al [18].

$$\begin{array}{l} REE\;(kcal/day)=(1.106\times \;{VCO}^{2}+3.941\times {VO}^{2})\times 1.44\\ RQ={VCO}^{2}÷{VO}^{2}\\ Protein\;Oxidation\;(PRO\;Ox)\;(g/day)=(0.15\times REE)÷16.74\\ Carbohydrate\;Oxidation\;(g/day)=[(4.55\;\times \;{VCO}^{2}-3.21\;\times \;{VO}^{2})\times 1.44]-0.459\times PRO\;Ox\\ Fat\;Oxidation\;(g/day)=[(1.67\times {VO}^{2}-1.67\;\times \;{VCO}^{2})\times 1.44]-0.307\times PRO\;Ox \end{array}$$

The changes over cold exposure in REE and substrate metabolism were calculated using the warm period (WP) as baseline.

#### Skin temperature and thermal comfort perception

Skin temperature was measured with 26 iButtons (adapted from Schellen et al [19]) (DS-1922 L, Thermochron; resolution: 0.0625°C; Maxim, Dallas, USA) attached to the skin on different body sites (Supporting information, S1 Fig). Skin temperature was recorded at 1-minute intervals and the mean [20], proximal [21], and distal skin temperature [22] were calculated. Furthermore, we calculated a peripheral gradient (forearm minus fingertip) [23] for each side of the body as a proxy of peripheral vasoconstriction. We also calculated the difference between the skin temperature of the right side of the chest and the supraclavicular zone as a proxy of BAT activity [24], and the whole-body gradient (distal minus proximal skin temperature) [21]. Equations used to determine the skin temperature parameters are shown in S1 Table. The analysis of all data recorded by the iButtons and the calculation of equations were carried out with the Temperatus software (http://profith.ugr.es/temperatus).

We used visual analogue scales to assess the thermal comfort perception, where 0 mm was “not cold at all” and 100 mm was “maximum tolerable cold” in both warm and cold conditions. The participants reported their thermal comfort perception in the whole body, as well as in their hands, feet, abdomen, and supraclavicular zone.

#### Superficial muscle activity (Surface electromyography)

Surface electromyography (EMG) wireless electrodes (Trigno Wireless Delsys EMG System, Boston, Massachusetts, USA) were placed on eight muscles (*Vastus Medialis*, *Vastus Lateralis*, *Rectus Femoris*, *Rectus Abdominis*, *Pectoralis Major*, *Deltoid*, *Trapezius*, *and Sternocleidomastoid*) on the right side of the body, following the current recommendations [25]. Raw EMG signals were amplified at a gain of 909 (differential amplifier, 20–450 Hz) and sampled at 2 kHz. A raw EMG data analysis was performed using Matlab (Version R2015a, The Mathworks, Natick, Massachusetts, USA). EMG signals were band-pass-filtered (20–500 Hz, 4th order zero-lag Butterworth filter), and the mean EMG root mean square (RMS) (mV) with a 50-ms moving rectangular window was calculated to determine activation throughout the assessments. The raw EMG RMS (mV) data were used for within-muscle changes in electrical activity comparisons throughout the cold exposure. The number of EMG activity bursts was also quantified to provide burst shivering rate (BSR) (bursts min^-1^) as a measure of the possible muscle activation during cold exposure. A shivering burst was defined as an EMG RMS period lasting ≥ 0.2 s at an amplitude greater than the intensity threshold and with a minimum inter-burst duration ≥ 0.75 s [26]. Intensity threshold was calculated by first averaging the EMG RMS activity and then averaging all EMG RMS values remaining above. The periods comprising voluntary movement were excluded from the analysis. The measurements were carried out in 6 out of the 11 participants.

#### Hemodynamics of the forearm and abdominal regions

We used a near infrared spatial resolved spectroscopy (NIR_SRS_) device (Portamon, Artinis Medical System, the Netherlands), a dual wavelength continuous system which simultaneously combines the modified Beer-Lambert and spatial resolved spectroscopy. We attached one device to the left ventral forearm (as representative of muscle tissue), in the medial point between the wrist and elbow joint, and another one to the left side of the abdomen (as representative of subcutaneous white adipose tissue), 2 cm from the umbilicus in the horizontal axis. Portamon provides the absolute value of tissue saturation index expressed as percentages (TSI%), and the relative changes in the concentration of total haemoglobin (ΔtHb), oxy-haemoglobin (ΔO2Hb), and deoxy-haemoglobin (ΔHHb), expressed in μmol.

Portamon light sources in both positions were situated at 30, 35, and 40 cm from the receptor, which allows a measurement of approximately 4 cm^3^ of volume and a penetration depth of approximately 2 cm [27]. Moreover, we assumed constant oxygen independent light losses due to scattering in tissue. A differential pathlength factor of 4 was established for the forearm [28,29] and for the abdomen (arbitrary value). The sample rate was set at 10 Hz and data were analysed with the Oxysof software (Portamon, Artinis Medical System, the Netherlands).

#### Heart rate variability

We used a Polar RS800CX (Polar Electro Oy, Kempele, Finland) heart rate monitoring system at 1000 Hz-frequency. The Polar RS800CX wirelessly receives heart rate data from a chest strap worn by participants. The data were analysed with Kubios HRV, version 2.2 software (Kuopio, Finland). We selected a low artefact correction level and applied smooth priors filter method with a lambda = 500 to remove trend components. Since the RR interval time series is an irregularly sampled series, a cubic spline interpolation rate of 4 Hz was used to convert the RR series into equidistantly sampled. Then, frequency bands were established at 0–0.04 Hz (very low frequency), 0.04–0.15 Hz (low frequency), and 0.15–0.4 (high frequency), and the spectrum estimation was calculated with the Fast Fourier Transformation (window width = 256 s, window overlap = 50%). We deleted the time intervals which did not meet a normal distribution, were unimodal, or presented outliers. The time and frequency domain parameters of heart rate variability were calculated. Concerning time domain parameters, we measured the mean length of all RR intervals, the percentage of consecutive normal RR intervals differing more than 50 ms, the square root of the mean squared sum of the differences of successive NN intervals, and the standard deviation of all RR length intervals. Regarding the frequency domain parameters, we measured the absolute and normalized power of high frequency and low frequency, as well as the low-high frequency ratio. High frequency seems to be an indicator of the parasympathetic nervous system tone, whereas low frequency is thought to be controlled by both sympathetic and parasympathetic systems [30–33]. Low frequency-high frequency ratio has been suggested as a measurement of sympathovagal balance [34], although it remains unclear [35].

#### Body composition

Body fat and lean mass were measured by Dual Energy X-ray Absorptiometry (HOLOGIC, QDR 4500 W) 48 hours after the completion of measurements. We also measured adipose tissue thickness at the abdomen and the forearm in triplicate using a skinfold caliper (British Indicators Ltd, UK). The measurements were taken at the same place where the Portamon diodes were placed, since adipose tissue thickness affects in vivo NIRS measurement [36].

### Statistical analysis

The data were analysed in 4 time periods (Fig 1): (i) WP; (ii) 31% and (iii) 64% of the individual’s cold exposure time until shivering occurred; and (iv) shivering threshold (ST). We used several temperature points along the mild cold exposure (31% and 64% of cold exposure and ST) to have a representative period of each third of the individualized cooling protocol. Measurements in each period were an average of 5 minutes. In the WP, we analysed the data of skin temperature, hemodynamics of the forearm and abdominal regions, and heart rate variability recorded between minutes 36–40. For REE and substrate metabolism analyses, we selected the 5 continuous minutes with the lowest mean coefficient of variance of oxygen consumption and carbon dioxide production volume, respiratory quotient, and minute ventilation between minutes 21–40. Similarly, the 5-minute period selected to analyse EMG was the one with the most stable values between minutes 31–40 during the WP. In the cold period, we analysed the 5 minutes immediately after the 31% or 64% of the individual’s time exposed to cold until shivering occurred. Finally, the ST period comprised the previous 5 minutes to shivering onset.

The changes over time in data of normally distributed variables (REE and substrate metabolism, skin temperature, thermal comfort perception, EMG RMS, hemodynamics of the forearm and abdominal regions, and heart rate variability) were analysed with a repeated measures analysis of variance (ANOVA). Pairwise comparisons were performed using the Bonferroni post-hoc tests. The changes over time in EMG BSR were analysed with Friedman Test, since they did not follow a normal distribution. Adjusted significance was chosen for Friedman test. The level of significance was set at P<0.05. Statistical Package for the Social Sciences (SPSS, version 22) was used to perform the statistical analysis (IBM, NewYork, USA).

## Results

**Fig 2:**
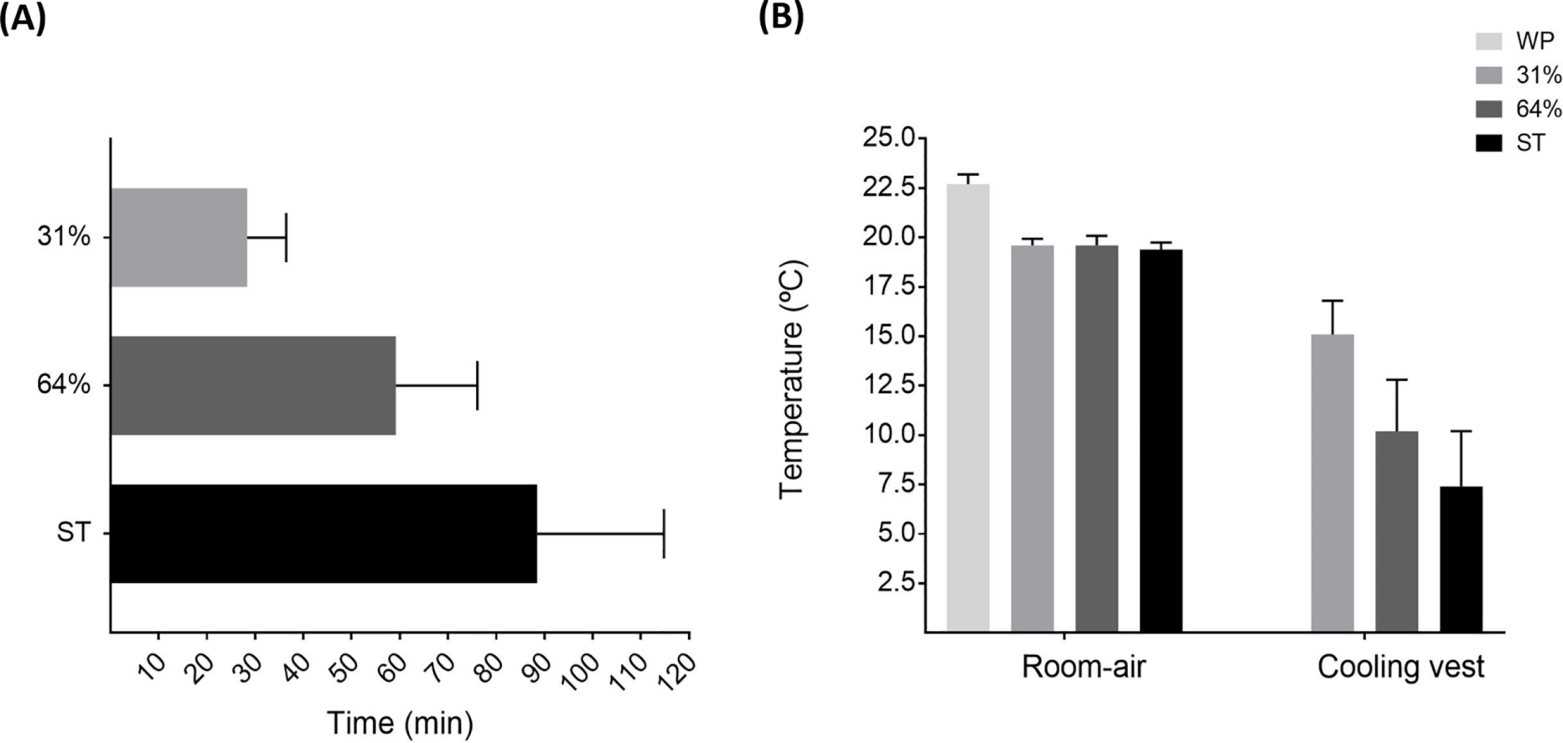
**Mean time of the study periods (A), and room-air and cooling vest temperature (B).** Values are mean ± standard deviation. ST: shivering threshold, WP: warm period, 31% and 64%: percentage of the individual’s time exposed to cold until shivering occurred.

Mean time ± standard deviation at which 31% and 64% of cold exposure and ST started was 28 ± 8 min, 59 ± 17 min, and 88 ± 26 min, respectively, after the beginnig of the cold exposure (Fig 2A). Fig 2B shows the room air and cooling vest temperature across periods.

### Resting energy expenditure and substrate metabolism

**Fig 3:**
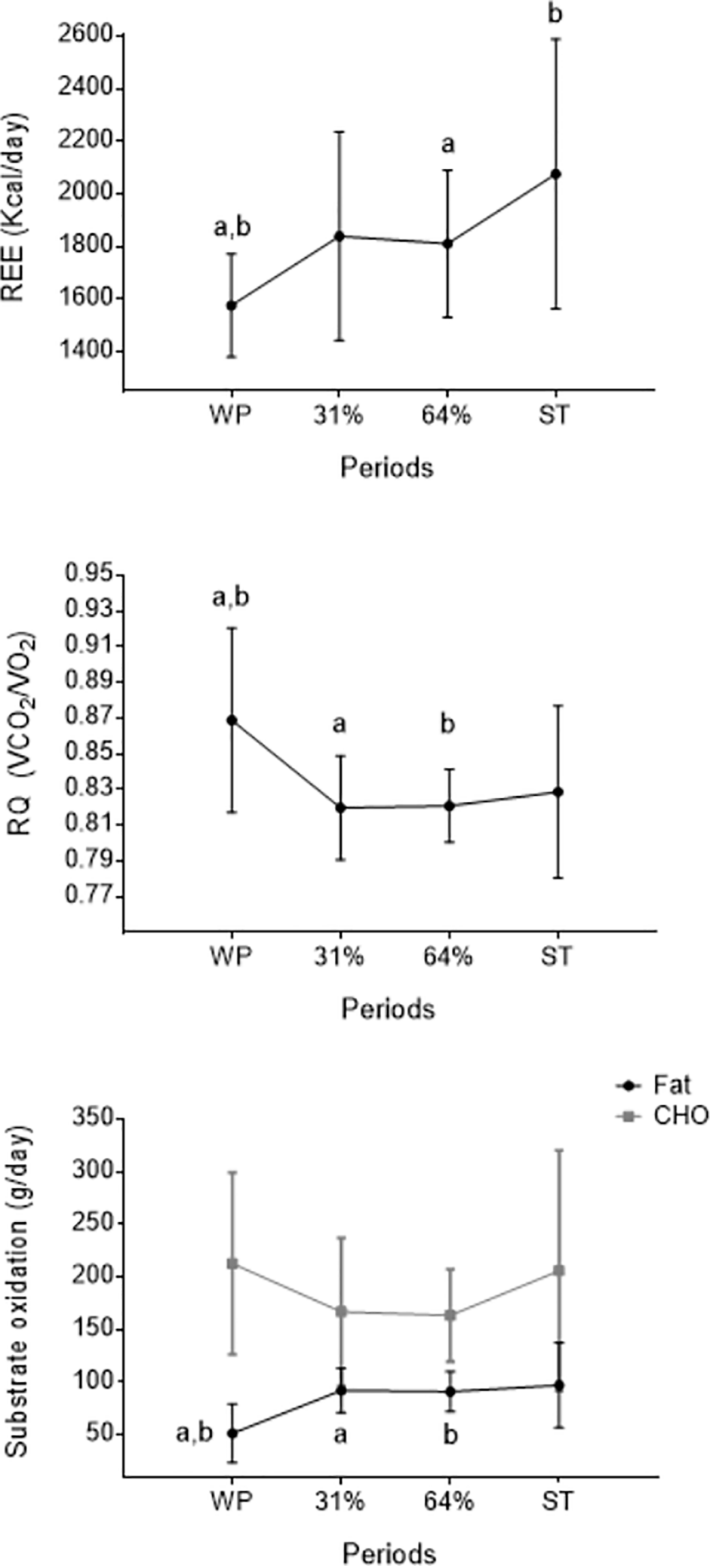
**Resting energy expenditure (A), respiratory quotient (B) and substrate metabolism (C) across study periods.** Values are mean ± standard deviation (n = 10). Repeated measures analysis of variance was performed, using Bonferroni post-hoc tests for pairwise comparisons. Common letters show significant differences (P≤0.05) between two specific periods. CHO: carbohydrates, REE: resting energy expenditure, RQ: respiratory quotient, ST: shivering threshold, WP: warm period, 31% and 64%: percentage of the individual’s time exposed to cold until shivering occurred.

REE increased from WP to 31% [mean difference (95% confidence interval): 263 kcal/day (24, 551), P = 0.078] and 64% [235 kcal/day (47, 423), P = 0.014] of cold exposure and ST [500 kcal/day (64, 936), P = 0.023; respectively] (Fig 3A). However, REE did not show any significant change from 31% and 64% of cold exposure to ST (P>0.05). Fat oxidation increased from WP to 31% and 64% of cold exposure [41 g/day (14, 68), P = 0.004; 40 g/day (13, 67), P = 0.005; respectively], whereas there was no change in carbohydrates oxidation (P>0.05, Fig 3B and 3C).

### Skin temperature and thermal comfort perception

#### Skin temperature

**Fig 4:**
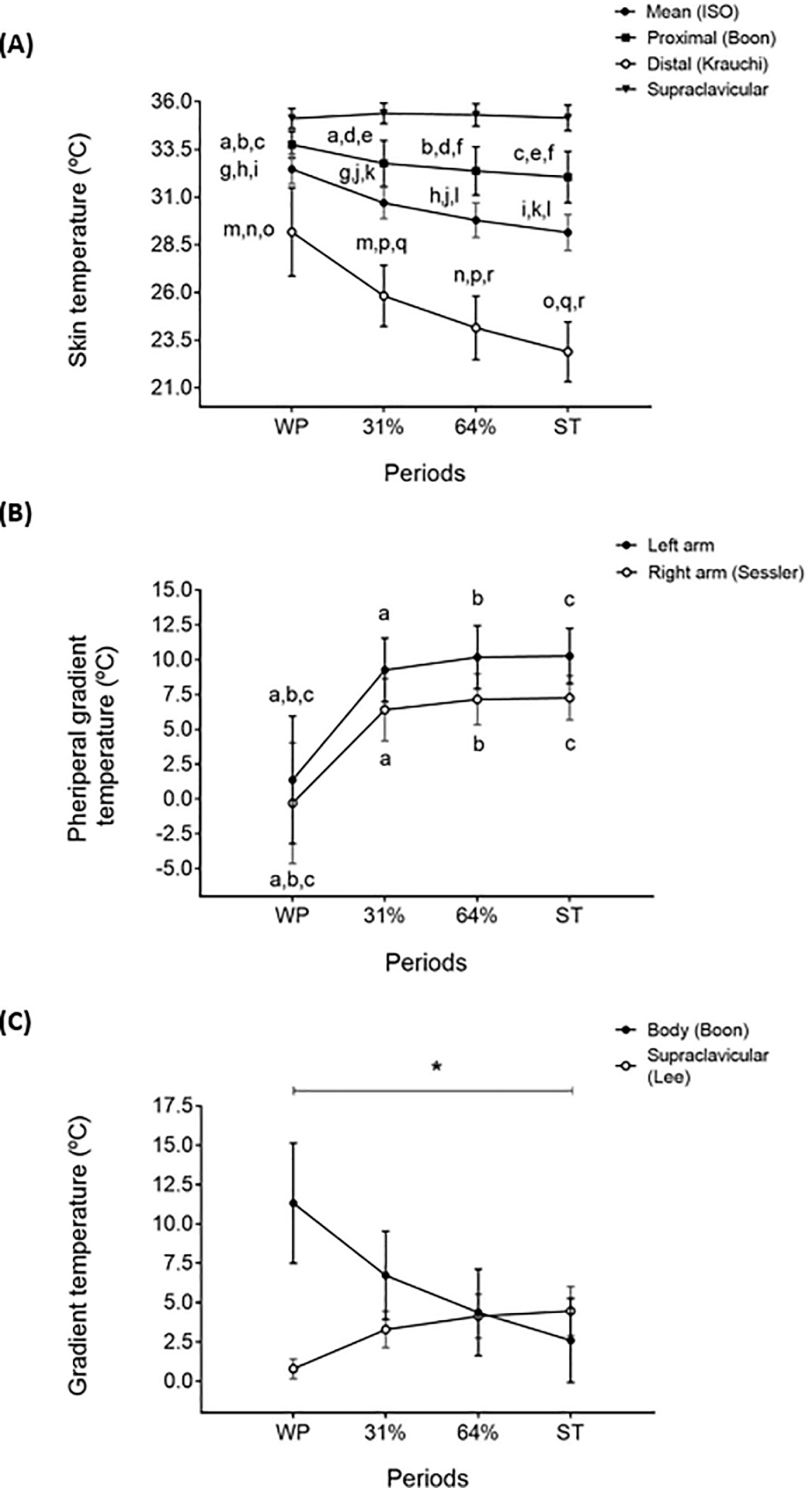
Skin temperature and body gradients across study periods. Panel (**A)**: skin temperature, Panel (**B**): proxies of peripheral vasoconstriction in both arms, Panel (**C**): body and supraclavicular skin temperature gradients. Values are mean ± standard deviation. Repeated measures analysis of variance was performed, using Bonferroni post-hoc tests for pairwise comparisons. Common letters show significant differences (P<0.05) between two specific periods. Symbol * shows significant differences among all periods (P<0.05). ST: shivering threshold, WP: warm period, 31% and 64%: percentage of the individual’s time exposed to cold until shivering occurred.

The mean, proximal, and distal skin temperature significantly decreased across all periods (all P≤0.05), whereas the right supraclavicular skin temperature did not show any significant change (P>0.05) (Fig 4A). The peripheral gradient (forearm minus fingertip) increased in both arms from WP to 31% and 64% of cold exposure and ST (all P≤0.001) (Fig 4B). Moreover, the supraclavicular gradient increased across all periods, whereas the whole-body gradient decreased (all P<0.05, all P≤0.001, respectively; Fig 4C).

#### Thermal comfort perception

**Fig 5:**
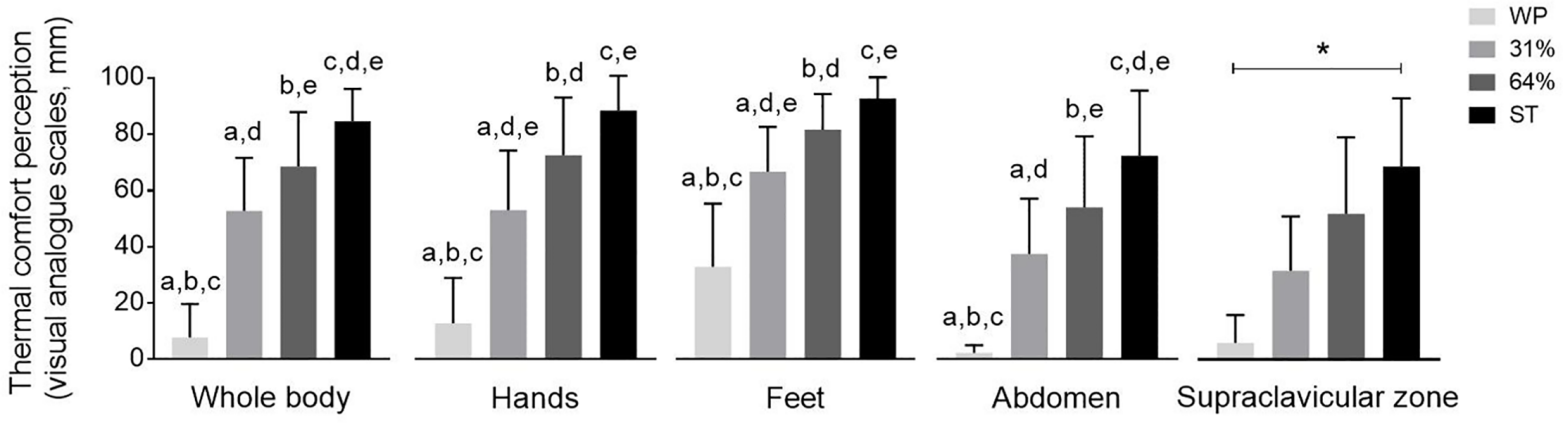
Thermal comfort perception measured by visual analogue scales across study periods. Visual analogue scales measured thermal comfort from “no cold at all” (= 0 mm) to “maximum tolerable cold” (= 100 mm). Values are mean ± standard deviation (n = 11). Repeated measures analysis of variance was performed, using Bonferroni post-hoc tests for pairwise comparisons. Common letters show significant differences between two specific periods (P<0.05). Symbol * shows significant differences among all time periods (P<0.05). ST: shivering threshold, WP: warm period, 31% and 64%: percentage of the individual’s time exposed to cold until shivering occurred.

A significant increase in thermal discomfort was observed in the whole body and in each body part reported from WP to 31% and 64% of cold exposure and ST (all P<0.05, Fig 5).

### Superficial muscle activity (EMG)

**Fig 6:**
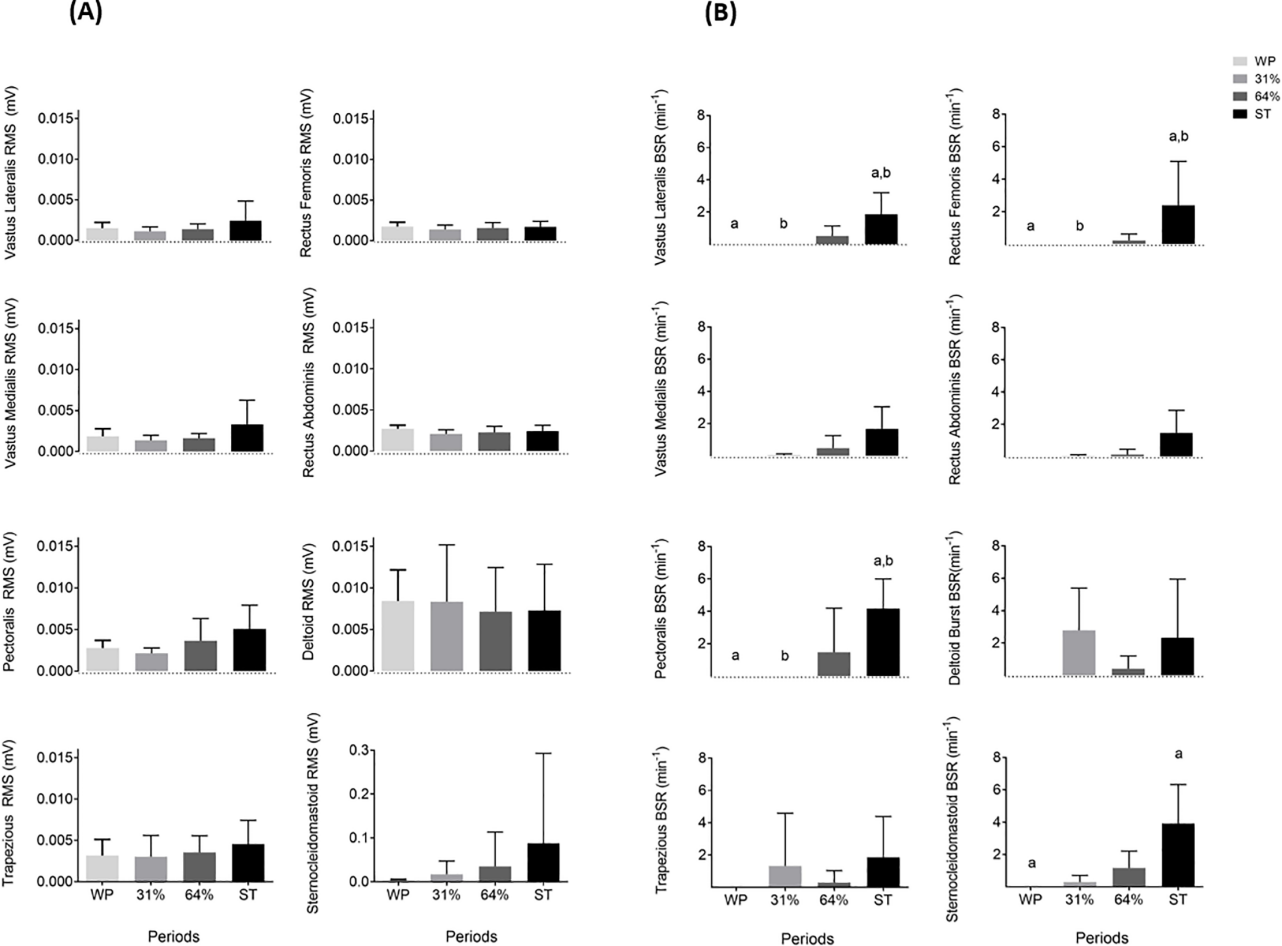
Electrical muscle activity (mV) and burst shivering rate (min^-1^) of eight different muscles across study periods. Panel (**A**): root mean square, Panel (**B**): burst shivering rate. Values are mean ± standard deviation (n = 6). Repeated measures analysis of variance (Bonferroni post-hoc tests) and Friedman test (adjusted significance) were respectively performed for EMG RMS and EMG BSR. Common letters show significant differences between periods (P < 0.05). BSR: burst shivering rate, RMS: root mean square, ST: shivering threshold, WP: warm period, 31% and 64%: percentage of the individual’s time exposed to cold until shivering occurred.

RMS did not show significant differences across periods in any muscle (P > 0.05). BSR in *Vastus Lateralis*, *Rectus Femoralis*, *and Pectoralis* showed an increase from WP and 31% of cold exposure to ST (all P<0.05). BSR of *Sternocleidomastoid* increased from WP to ST (P<0.05). The results (n = 6) are shown in Fig 6.

### Hemodynamics of the forearm and abdominal regions (NIR_SRS_ parameters)

**Fig 7:**
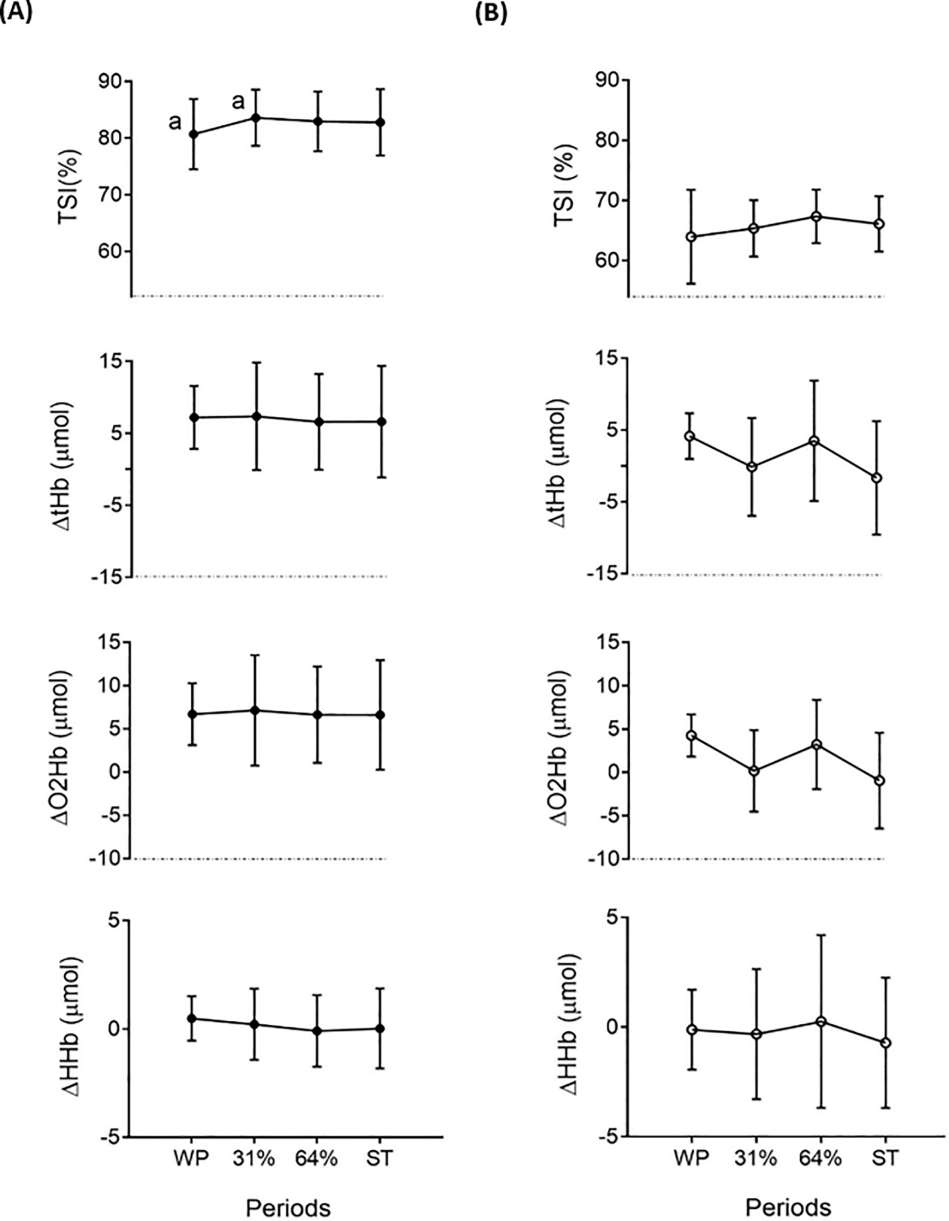
Tissue saturation index (%) and relative changes in the concentration of total haemoglobin (ΔtHb), oxy-haemoglobin (ΔO_2_Hb), and deoxy-haemoglobin (ΔHHb) in the abdominal and forearm regions across study periods. Panel (**A**): abdominal region, Panel (**B**): forearm region. Values are mean ± standard deviation (n = 9). Repeated measures analysis of variance was performed, using Bonferroni post-hoc tests for pairwise comparisons. Common letters show significant differences between two specific periods (P<0.05). ST: shivering threshold, WP: warm period, 31% and 64%: percentage of the individual’s time exposed to cold until shivering occurred.

TSI% increased from WP to 31% of cold exposure [2.89% (5.5, 0.3), P = 0.032] in the abdominal region (Fig 7), whereas no differences were found in ΔtHb, ΔO_2_Hb, and ΔHHb across periods (P>0.05). No changes were observed in TSI%, ΔtHb, ΔO_2_Hb, and ΔHHb in the forearm region through all periods (P>0.05, Fig 7).

### Heart rate variability

**Fig 8:**
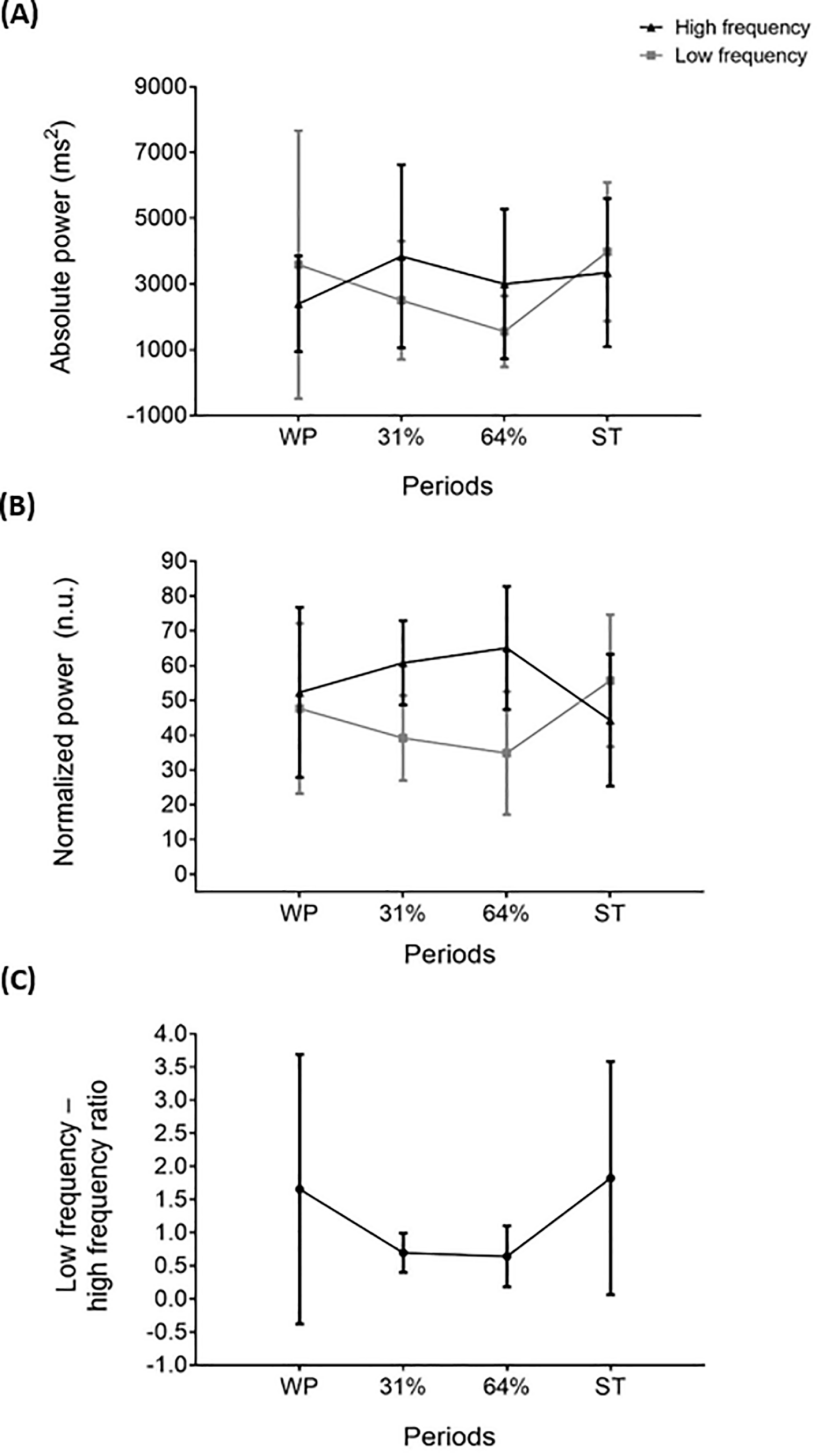
Frequency domain parameters of heart rate variability across study periods. Panel (**A**): absolute power, Panel (**B**): normalized power, Panel (**C**): low frequency—high frequency ratio. Values are mean ± standard deviation (n = 7). Repeated measures analysis of variance was performed, using Bonferroni post-hoc tests for pairwise comparisons. No significant differences were found across study periods (P>0.05). ST: shivering threshold, WP: warm period, 31% and 64%: percentage of the individual’s time exposed to cold until shivering occurred.

All frequency domain parameters of heart rate variability were similar across periods (P>0.05, Fig 8). Furthermore, time domain parameters did not change (P>0.05, S2 Table).

## Discussion

In the present study, we extend previous findings on the potential of mild cold exposure to increase REE and to prompt fat-oxidative metabolism in young lean men. As shivering came closer, skin temperature (mean, proximal and distal) and thermal comfort perception decreased, except the supraclavicular skin temperature, which did not change. Furthermore, superficial muscle activation increased in the ST, when shivering was reported by the participants and visually determined by the researchers. It is noteworthy that the largest physiological changes occurred between WP and 31% (≈ 30 minutes) of the individual’s cold exposure time, when the participants reported less discomfort.

### Shivering threshold

Most studies examining the effect of acute cold exposure on human metabolism have used a standardized and non-individualized cooling protocol (e.g. similar duration, intensity, and type of cold stimulus for every participant), in order to make experiments replicable. However, when non-individualized cooling protocols are applied, inter-individual differences are not considered, which is likely to affect the cold induced physiological responses. Furthermore, given the fact that non-shivering thermogenesis shows a high inter-individual variability [1], to individualize the cooling protocol to every participant becomes relevant.

The complex nature of shivering makes it difficult to determine its onset and regulation in humans [37]. In this experiment, “shivering threshold” was used as the end-point of the cooling protocol, in which shivering was self-reported by the participants and visually determined by the researchers. The use of a self-reported shivering threshold might not be considered as a valid method to establish shivering onset, since conscious thermal perception and localization are regulated by the thalamus and cerebral cortex, whereas shivering is controlled by the preoptic area of the hypothalamus [5,37]. In addition, the visual determination of shivering threshold by researchers is not an objective measurement. Hence, the shivering threshold was only considered an indicator of shivering onset that allowed us to individualize the cooling protocol for every participant. Nevertheless, to the best of our knowledge, no studies have compared yet whether the use of a subjectively determined shivering threshold differs from the onset of shivering determined by EMG. To note also is that shivering might be a combination of both voluntary and involuntary muscular contractions, and consequently EMG could also present inaccuracies in determining the onset of shivering. Furthermore, although other methods such as temperature clamping have been proposed [38], the use of shivering threshold has been extensively used and accepted as a valid method to maximize non-shivering thermogenesis and activate BAT [21,39–43]. Most important, the participants from this study underwent the same cooling protocol 48 hours after, replicating all conditions, and the time at which shivering threshold took place did not change (unpublished observations). Consequently, despite its limitations, shivering threshold may a potential and reliable method to determine shivering onset.

### Resting energy expenditure and substrate metabolism

We observed a mean total increase in REE of 31.7% since the participants were exposed to mild cold until they shivered, which concurs with previous literature [1,2]. The increase in REE from WP to 31% of cold exposure accounted for a large part of total REE increase (16.7%). Regarding substrate metabolism, previous studies [44,45] have shown that mild cold exposure is likely to induce an increase in fat oxidation, whereas carbohydrate oxidation decreases. In line with these reports, we observed a large increase in fat oxidation (72.6%) from WP to 31% of cold exposure, whereas CHO remained unchanged along the cold exposure. To note is that a plateau on REE and substrate metabolism was observed from 31% to 64% of cold exposure, which could indicate an initial adaptation to cold or a shift in the metabolic pathway. Taking all findings together, it seems that non-shivering thermogenesis accounted for a large part of CIT, especially during the initial moments of cold exposure, that is around the first 30 minutes in our cooling protocol. Furthermore, superficial muscle increased its shivering activity during the last minutes of cooling, clearly contributing to the increase of REE. Although shivering thermogenesis has been reported to increase REE up to 5 times [3,4], we observed a lower increase in REE since our cooling protocol was designed to finish at the onset of shivering.

### Skin temperature and thermal comfort perception

Skin temperature decreased in most of the measured body anatomical points along the cold exposure, but the skin temperature of the supraclavicular zone did not. The skin temperature at the supraclavicular zone is an indirect marker of BAT activity or volume during cold exposure [21,46,47]. Therefore, it is plausible that BAT accounted for a part of non-shivering thermogenesis. This assumption is supported by the increase of the supraclavicular gradient, which is an indirect marker of the heat loss capacity of the supraclavicular zone [24]. In addition, an increase in the gradient of the right arm used as a proxy of peripheral vasoconstriction [23] above 4°C indicated that a cold-induced peripheral vasoconstriction took place from WP to 31% of cold exposure [48,49]. The largest skin temperature changes were observed from WP to 31% of cold exposure, the period of cold exposure in which the participants felt less discomfort either in the whole body or in each body part.

### Superficial muscle activity

As expected, a general increase in superficial muscle activation was observed as shivering came closer, noticing an increase in the BSR from the WP and 31% of cold exposure to ST. Although changes were not statistically significant (P>0.05), most muscles had a minimum or noticeable burst shivering rate during 64% of cold exposure when shivering was not reported by participants or visually detected by researchers. This finding suggests that tests designed to analyse the non-shivering thermogenesis and that are not controlled by electromyography are partially influenced by superficial muscle activity. The increase of CIT across cold exposure may also be explained by the shivering activity of deeper central-located muscles, which was not determined in this study [15]. Further studies should quantify both superficial and deep muscle activity by electromyography during cold exposure to understand better the actual contribution of muscle to CIT. Of note is also that deltoid showed a different trend in shivering to the rest of muscles, showing a high muscle BSR during the 31% of cold exposure. This trend seemed to be cause of voluntary movement by the participants rather than the thermal strain caused by the cooling protocol.

### Hemodynamics of forearm and abdominal regions (NIR_SRS_ parameters)

The use of NIRS to study changes in oxidative metabolism during cold exposure is relatively recent [50,51]. Several authors have used NIRS parameters such as regional blood oxygen saturation as a proxy of tissue oxidative metabolism [50], and total haemoglobin as an index of blood volume or tissue vasculature [51]. However, the use of different types of devices and the lack of quantification hampers inter-studies comparisons. In the present study, TSI% (an indicator of the oxygen saturation of regional vasculature) increased from WP to 31% of cold exposure in the abdominal region (used as a proxy of subcutaneous white adipose tissue oxygenation), whereas ΔtHb (an index of blood volume) did not change. Consequently, since blood volume was constant and oxygen saturation of regional vasculature increased, abdominal subcutaneous white adipose tissue oxidative metabolism decreased from WP to 31% of cold exposure. This finding suggests that subcutaneous white adipose tissue is not involved in the observed increase of REE during cold exposure.

### Heart rate variability

Cold exposure increases sympathetic nervous system activity, inducing norepinephrine release and prompting a range of physiological responses as well as brown adipose tissue activation [52–54]. Consequently, it was of interest to determine whether heart rate variability parameters will change in response to mild cold, and more specifically, whether low frequency-high frequency ratio would increase since it has been proposed as an indirect marker of sympathovagal balance [34]. Studies focusing on the effect of acute cold exposure over heart rate variability are scarce. Several experiments have suggested that cold exposure is related to changes in the autonomic sympathetic response [55] or to a higher sympathetic nervous system predominance over parasympathetic system in humans [56]. Nevertheless, in the present study we did not find significant changes in any heart rate variability parameter, probably due to the high inter-individual variability of these parameters. Furthermore, there is still controversy regarding whether the low frequency-high frequency ratio actually reflects sympathovagal balance [35], which could explain why no changes in this parameter were either observed.

### Comprehensive insight

There is controversy regarding the underlying mechanisms of CIT during mild cold exposure. Whether shivering or non-shivering thermogenesis act together or independently, and to what extent each component contributes to CIT still remains unclear [1,2]. Several authors have proposed BAT as one of the main mediators of non-shivering thermogenesis [13,14], whereas others suggest that muscle is more predominant [7,15]. It has also been postulated that BAT and muscle contribute synergistically to non-shivering thermogenesis [57]. Less is known, however, about the role of subcutaneous white adipose tissue over non-shivering thermogenesis. Despite the need of more evidence, we observed that the largest increase of non-shivering thermogenesis in humans would normally happen during initial moments of cold exposure, as we observed in the 31% of cold exposure. Since BAT is mainly fuelled by triglycerides obtained by BAT intracellular lipolysis and plasma non esterified fatty acids [12,13,15,58], the large increase in fat oxidation (72.6%) from WP to 31% of cold exposure suggests that BAT is active. The decrease of skin temperature in most measured anatomical points, but not in the supraclavicular zone, supports this assumption. These findings add further evidence indicating that BAT acts as a non-shivering thermogenesis effector during mild cold exposure [13].

However, as previously mentioned, the skeletal muscle has also been postulated as a possible contributor to non-shivering thermogenesis and might account for a part of non-esterified fatty acids clearance, especially in proximal deep muscles such as “Longus colli” [15]. Skeletal muscle seems to mainly contribute to glucose turnover, even when shivering is minimized [15]. Nevertheless, the increase in fat oxidation is such that it is not plausible to think that BAT itself could account for all of it. This leads to the idea that muscle increases its fat oxidative metabolism via mitochondrial uncoupling [7] or by low intensity shivering [59]. Hence, BAT and muscle might contribute to non-shivering thermogenesis synergistically, especially at the beginning of mild cold exposure. In addition, the plateau observed in REE and substrate metabolism from 31% to 64% of cold exposure, followed by changes in the trends of substrate metabolism (despite not being statistically significant) might indicate a shift in the metabolic pathway. This metabolic shift could reflect changes in the relative contribution of BAT and skeletal muscle to non-shivering thermogenesis [5], being BAT contribution higher at the beginning when thermal stress was lower, and increasing muscle contribution (and consequently CHO oxidation) as shivering came closer.

UCP-1 and other brown fat cell genes, characteristic of beige adipocytes, have shown to be prominent in subcutaneous white adipose tissue depots of rodents [60]. Thus, we hypothesized that subcutaneous white adipose tissue would increase its oxidative metabolism during cold exposure as shown by Muzik et al. [50]. However, the oxidative metabolism of the abdominal region seemed to decrease. This might be explained by the fact that abdominal subcutaneous white adipose tissue depots seem to present a higher resistance to browning [60–62] or that they mostly represent depots of pure white adipocytes. In any case, caution must be paid since NIRS measurement is only a proxy of oxidative metabolism and has not been validated yet in subcutaneous white adipose tissue.

Despite the fact that mild cold exposure has been suggested to play a central role counteracting obesity at long term [2,11,63], we only observed a small increase in CIT (~20.83 kcal/h), and consequently its contribution to cause a negative energy balance may be negligible. Nevertheless, mild cold exposure seems to have an important role in counteracting body fat accumulation and in obesity related comorbidities [7–10]. Finally, it is noteworthy that the largest physiological changes in metabolism and thermoregulation occurred during the initial moments of the cold exposure. This occurred specifically from WP to 31% (first ≈ 30 minutes) of the cold exposure, when the participants showed the lowest discomfort perception across cold exposure. This finding provides practical guidelines for future uses of mild cold as a health promoter stimulus, so that the effect of cold in a short time can be maximized while increasing adherence.

A limitation to consider in the present study is that despite the use of the supraclavicular skin temperature as a surrogate marker of BAT activity or volume [21,46,47], we cannot exclude that it is in fact registering the temperature of large blood vessels (i.e. aorta) close to the skin in this area. In addition, we did not measure core temperature, which might have provided useful information to determine whether mild cold exposure actually elicited any thermal benefit. It is also noticeable that the use of superficial EMG only allowed us to study the contribution of superficial muscles to CIT. Regarding NIR_SRS_ parameters, the in vivo scattering properties of the biological tissues and the unknown contribution of myoglobin to the NIRS signal were inherent limitations. On the other hand, we provide a comprehensive insight of the physiological changes that occur during an acute bout of mild cold exposure, using an individualized cooling protocol designed to determine the shivering threshold. We used several temperature points along the cold exposure (31%, 64% of cold exposure and ST) to better analyse the physiological changes through the whole spectrum of non-shivering thermogenesis. This fact is noteworthy, since experiments normally consider only 2 different conditions (warm and cold), lacking important information [1].

In conclusion, non-shivering thermogenesis seems to develop an important role increasing CIT during mild cold exposure, being accompanied by a higher fat oxidative metabolism. Both skeletal muscle and BAT might contribute synergistically to NST increase, whereas the subcutaneous white adipose tissue does not seem to be a key player. Furthermore, we observed that the largest physiological changes occurred during the first 30 minutes of cold exposure, when the participants felt less discomfort during the cold exposure. However, more evidence is needed to understand the underlying mechanisms of non-shivering thermogenesis during mild cold exposure.

## Supporting information

S1 Fig Body anatomical points where the iButtons were attached to the skin. 26 different positions can be distinguished. Panel (**A**): distribution of the iButtons over the whole body, Panel (**B**): distribution of the iButtons on the right clavicular sites.
(PDF)

S1 Table Equations used to measure skin temperature. Table adapted from Martinez-Tellez et al. [64].
(PDF)

S2 Table Time domain parameters of heart rate variability rate across study periods. Values are mean ± standard deviation (n = 7). Repetead measures analysis of variance was performed, using Bonferroni post-hoc tests for pairwise comparisons. No significant differences were observed across periods (P>0.05). Mean RR: mean length of all RR intervals, pNN50: percentage of consecutive normal RR intervals differing more than 50 ms, RMSSD: square root of the mean squared sum of the differences of successive NN intervals, SDNN: standard deviation of all RR legnth intervals, ST: shivering thereshold period, WP: warm period, 31% and 64%: percentage of the individual’s time exposed to cold until shivering occurred.
(PDF)

## References

[1] Brychta RJ, Chen KY. Cold-induced thermogenesis in humans. Eur J Clin Nutr. 2017 3 23;71(3):345–52. doi: 10.1038/ejcn.2016.223 27876809 10.1038/ejcn.2016.223 PMC6449850

[2] van Marken Lichtenbelt WD, Schrauwen P. Implications of nonshivering thermogenesis for energy balance regulation in humans. AJP Regul Integr Comp Physiol. 2011 8 1;301(2):285–96. 10.1152/ajpregu.00652.2010 21490370

[3] Eyolfson DA, Tikuisis P, Xu X, Weseen G, Giesbrecht GG. Measurement and prediction of peak shivering intensity in humans. Eur J Appl Physiol. 2001 2 16;84(1–2):100–6. doi: 10.1007/s004210000329 11394237 10.1007/s004210000329

[4] Haman F. Shivering in the cold: from mechanisms of fuel selection to survival. J Appl Physiol. 2006 5 1;100(5):1702–8. doi: 10.1152/japplphysiol.01088.2005 16614367 10.1152/japplphysiol.01088.2005

[5] Blondin DP, Tingelstad HC, Mantha OL, Gosselin C, Haman F. Maintaining thermogenesis in cold exposed humans: Relying on multiple metabolic pathways. Compr Physiol. 2014;4(4):1383–402. doi: 10.1002/cphy.c130043 25428848 10.1002/cphy.c130043

[6] Boon MR, van Marken Lichtenbelt WD. Brown Adipose Tissue: A Human Perspective. In: Handbook of experimental pharmacology. 2015 p. 301–19. 26003832 10.1007/164_2015_11

[7] Wijers SLJ, Schrauwen P, Saris WHM, van Marken Lichtenbelt WD. Human Skeletal Muscle Mitochondrial Uncoupling Is Associated with Cold Induced Adaptive Thermogenesis. Bartolomucci A, editor. PLoS One. 2008 3 12;3(3):e1777 doi: 10.1371/journal.pone.0001777 18335051 10.1371/journal.pone.0001777 PMC2258415

[8] Hanssen MJW, Hoeks J, Brans B, van der Lans AAJJ, Schaart G, van den Driessche JJ, et al Short-term cold acclimation improves insulin sensitivity in patients with type 2 diabetes mellitus. Nat Med. 2015;21(8):863–5. doi: 10.1038/nm.3891 26147760 10.1038/nm.3891

[9] Lee P, Smith S, Linderman J, Courville AB, Brychta RJ, Dieckmann W, et al Temperature-Acclimated Brown Adipose Tissue Modulates Insulin Sensitivity in Humans. Diabetes. 2014;63(11):3686–98. doi: 10.2337/db14-0513 24954193 10.2337/db14-0513 PMC4207391

[10] Palmer BF, Clegg DJ. Non-shivering thermogenesis as a mechanism to facilitate sustainable weight loss. Obes Rev. 2017;18(8):819–31. doi: 10.1111/obr.12563 28547916 10.1111/obr.12563

[11] Wijers SLJ, Saris WHM, Van Marken Lichtenbelt WD. Recent advances in adaptive thermogenesis: Potential implications for the treatment of obesity: Obesity Management. Obesity Reviews 3, 2009 p. 218–26. 19021870 10.1111/j.1467-789X.2008.00538.x

[12] Cannon B, Nedergaard J. Brown Adipose Tissue: Function and Physiological Significance. Physiol Rev. 2004 1 1;84(1):277–359. doi: 10.1152/physrev.00015.2003 14715917 10.1152/physrev.00015.2003

[13] Ouellet V, Labbé SM, Blondin DP, Phoenix S, Guérin B, Haman F, et al Brown adipose tissue oxidative metabolism contributles to energy expenditure during cold exposure in humans. J Clin Invest. 2012;122(2):545 doi: 10.1172/JCI60433 22269323 10.1172/JCI60433 PMC3266793

[14] Chen KY, Brychta RJ, Linderman JD, Smith S, Courville A, Dieckmann W, et al Brown Fat Activation Mediates Cold-Induced Thermogenesis in Adult Humans in Response to a Mild Decrease in Ambient Temperature. J Clin Endocrinol Metab. 2013 7;98(7):E1218–23. doi: 10.1210/jc.2012-4213 23780370 10.1210/jc.2012-4213 PMC3701264

[15] Blondin DP, Labbé SM, Phoenix S, Guérin B, Turcotte ÉE, Richard D, et al Contributions of white and brown adipose tissues and skeletal muscles to acute cold-induced metabolic responses in healthy men. J Physiol. 2015 2 1;593(3):701–14. doi: 10.1113/jphysiol.2014.283598 25384777 10.1113/jphysiol.2014.283598 PMC4324714

[16] Weir J. B. De V. New methods for calculating metabolic rate with special reference to protein metabolism. J Physiol. 1949;109(5):1–9. 15394301 10.1113/jphysiol.1949.sp004363 PMC1392602

[17] Frayn KN. Calculation of substrate oxidation rates in vivo from gaseous exchange. J Appl Physiol. 1983 8;55(2):628–34. doi: 10.1152/jappl.1983.55.2.628 6618956 10.1152/jappl.1983.55.2.628

[18] Schadewaldt P, Nowotny B, Strassburger K, Kotzka JJ, Roden M. Indirect calorimetry in humans: a postcalorimetric evaluation procedure for correction of metabolic monitor variability. Am J Clin Nutr. 2013 4 1;97(4):763–73. doi: 10.3945/ajcn.112.035014 23446893 10.3945/ajcn.112.035014

[19] Schellen L, Loomans MGLC, de Wit MH, Olesen BW, Lichtenbelt WD van M. The influence of local effects on thermal sensation under non-uniform environmental conditions—Gender differences in thermophysiology, thermal comfort and productivity during convective and radiant cooling. Physiol Behav. 2012;107(2):252–61. doi: 10.1016/j.physbeh.2012.07.008 22877870 10.1016/j.physbeh.2012.07.008

[20] ISO-standard 9886:2004. Ergonomics–Evaluation of thermal strain by physiological measurements, International Standards Organization, Geneva, Switzerland. Geneva, Switzerland.; 2004 p. 1–21.

[21] Boon MR, Bakker LEH, van der Linden RAD, Pereira Arias-Bouda L, Smit F, Verberne HJ, et al Supraclavicular skin temperature as a measure of 18F-FDG uptake by BAT in human subjects. PLoS One. 2014 6 12;9(6):e98822 doi: 10.1371/journal.pone.0098822 24922545 10.1371/journal.pone.0098822 PMC4055666

[22] Kräuchi K, Cajochen C, Möri D, Graw P, Wirz-Justice A. Early evening melatonin and S-20098 advance circadian phase and nocturnal regulation of core body temperature. Am J Physiol. 1997;272(4 Pt 2):R1178–88. 9140018 10.1152/ajpregu.1997.272.4.R1178

[23] Sessler DI, Olofsson CI, Rubinstein EH, Beebe JJ. The thermoregulatory threshold in humans during halothane anesthesia. Anesthesiology. 1988 6;68(6):836–42. 3377230 10.1097/00000542-198806000-00002

[24] Lee P, Ho KKY, Greenfield JR. Hot fat in a cool man: infrared thermography and brown adipose tissue. Diabetes, Obes Metab. 2011 1;13(1):92–3. 21114609 10.1111/j.1463-1326.2010.01318.x

[25] Hermens HJ, Freriks B, Disselhorst-Klug C, Rau G. Development of recommendations for SEMG sensors and sensor placement procedures. J Electromyogr Kinesiol. 2000 10;10(5):361–74. 11018445 10.1016/s1050-6411(00)00027-4

[26] Haman F, Legault SR, Weber J-M. Fuel selection during intense shivering in humans: EMG pattern reflects carbohydrate oxidation. J Physiol. 2004 4;556(1):305–13. 14742724 10.1113/jphysiol.2003.055152 PMC1664890

[27] Chance B, Nioka S, Kent J, McCully K, Fountain M, Greenfeld R, et al Time-resolved spectroscopy of hemoglobin and myoglobin in resting and ischemic muscle. Anal Biochem. 1988 11;174(2):698–707. 3239768 10.1016/0003-2697(88)90076-0

[28] Ferrari M, Wei Q, Carraresi L, De Blasi RA, Zaccanti G. Time-resolved spectroscopy of the human forearm. J Photochem Photobiol B. 1992;16(2):141–53. 1474423 10.1016/1011-1344(92)80005-g

[29] Duncan A, Meek JH, Clemence M, Elwell CE, Tyszczuk L, Cope M, et al Optical pathlength measurements on adult head, calf and forearm and the head of the newborn infant using phase resolved optical spectroscopy. Phys Med Biol. 1995;40(2):295–304. 7708855 10.1088/0031-9155/40/2/007

[30] Akselrod S, Gordon D, Ubel FA, Shannon DC, Berger AC, Cohen RJ. Power Spectrum Analysis of Heart Rate Fluctuation: A Quantitative Probe of Beat-to-Beat Cardiovascular Control. Science. 1981 7 10;213(July):220–2. 6166045 10.1126/science.6166045

[31] Saul JP, Rea RF, Eckberg DL, Berger RD, Cohen RJ. Heart-rate and muscle sympathetic-nerve variability during reflex changes of autonomic activity. Am J Physiol. 1990;258(3):h713–21. 2316686 10.1152/ajpheart.1990.258.3.H713

[32] Ori Z, Monir G, Weiss J, Sayhouni X, Singer DH. Heart rate variability. Frequency domain analysis. Cardiol Clin. 1992 8;10(3):499–537. 1504981

[33] Heart rate variability. Standards of measurement, physiological interpretation, and clinical use. Task Force of the European Society of Cardiology and the North American Society of Pacing and Electrophysiology. Eur Heart J. 1996;17:354–81. 8737210

[34] Pagani M, Lombardi F, Guzzetti S, Rimoldi O, Furlan R, Pizzinelli P, et al Power spectral analysis of heart rate and arterial pressure variabilities as a marker of sympatho-vagal interaction in man and conscious dog. Circ Res. 1986;59(2):178–93. 2874900 10.1161/01.res.59.2.178

[35] Billman GE. The LF/HF ratio does not accurately measure cardiac sympatho-vagal balance. Front Physiol. 2013;4:26 doi: 10.3389/fphys.2013.00026 23431279 10.3389/fphys.2013.00026 PMC3576706

[36] Van Beekvelt M, Borghuis M, Van Engelen B, Wevers R, Colier W. Adipose tissue thickness affects in vivo quantitative near-IR spectroscopy in human skeletal muscle. Clin Sci. 2001 7 1;101(1):21 11410110 10.1042/cs20000247

[37] Haman F, Blondin DP. Shivering thermogenesis in humans: Origin, contribution and metabolic requirement. Temperature. 2017;(May):1–10. 10.1080/23328940.2017.1328999 PMC5605160 28944268

[38] Blondin DP, Tingelstad HC, Noll C, Frisch F, Phoenix S, Guérin B, et al Dietary fatty acid metabolism of brown adipose tissue in cold-acclimated men. Nat Commun. 2017;8. 10.1038/ncomms14146 PMC5290270 28134339

[39] Chen KY, Cypess AM, Laughlin MR, Haft CR, Hu HH, Bredella MA, et al Brown Adipose Reporting Criteria in Imaging STudies (BARCIST 1.0): Recommendations for Standardized FDG-PET/CT Experiments in Humans. Cell Metab. 2016 8;24(2):210–22. doi: 10.1016/j.cmet.2016.07.014 27508870 10.1016/j.cmet.2016.07.014 PMC4981083

[40] Cypess AM, Haft CR, Laughlin MR, Hu HH. Brown fat in humans: Consensus points and experimental guidelines. Cell Metab. 2014;20(3):408–15. doi: 10.1016/j.cmet.2014.07.025 25185947 10.1016/j.cmet.2014.07.025 PMC4155326

[41] van der Lans AAJJ, Wierts R, Vosselman MJ, Schrauwen P, Brans B, van Marken Lichtenbelt WD. Cold-activated brown adipose tissue in human adults: methodological issues. AJP Regul Integr Comp Physiol. 2014;307(2):R103–13. 10.1152/ajpregu.00021.2014 24871967

[42] Vijgen GHEJ, Bouvy ND, Teule GJJ, Brans B, Schrauwen P, van Marken Lichtenbelt WD. Brown Adipose Tissue in Morbidly Obese Subjects. PLoS One. 2011;6(2):e17247 doi: 10.1371/journal.pone.0017247 21390318 10.1371/journal.pone.0017247 PMC3044745

[43] Sanchez-Delgado G, Martinez-Tellez B, Olza J, Aguilera CM, Labayen I, Ortega FB, et al Activating brown adipose tissue through exercise (ACTIBATE) in young adults: Rationale, design and methodology. Contemp Clin Trials. 2015;45:416–25. doi: 10.1016/j.cct.2015.11.004 26546068 10.1016/j.cct.2015.11.004

[44] Vosselman MJ, Brans B, Van Der Lans AAJJ, Wierts R, Van Baak MA, Mottaghy FM, et al Brown adipose tissue activity after a high-calorie meal in humans. Am J Clin Nutr. 2013 7 1;98(1):57–64. doi: 10.3945/ajcn.113.059022 23719558 10.3945/ajcn.113.059022

[45] Vosselman MJ, Hoeks J, Brans B, Pallubinsky H, Nascimento EBM, Van Der Lans AAJJ, et al Low brown adipose tissue activity in endurance-trained compared with lean sedentary men. Int J Obes. 2015;39(12):1696–702. 10.1038/ijo.2015.130 26189600

[46] van der Lans AAJJ, Vosselman MJ, Hanssen MJW, Brans B, van Marken Lichtenbelt WD. Supraclavicular skin temperature and BAT activity in lean healthy adults. J Physiol Sci. 2016 1 29;66(1):77–83. doi: 10.1007/s12576-015-0398-z 26420686 10.1007/s12576-015-0398-z PMC4676963

[47] Chondronikola M, Volpi E, Børsheim E, Chao T, Porter C, Annamalai P, et al Brown adipose tissue is linked to a distinct thermoregulatory response to mild cold in people. Front Physiol. 2016 4 19;7(APR):129 doi: 10.3389/fphys.2016.00129 27148068 10.3389/fphys.2016.00129 PMC4835478

[48] House JR, Tipton MJ. Using skin temperature gradients or skin heat flux measurements to determine thresholds of vasoconstriction and vasodilatation. Eur J Appl Physiol. 2002 11 1;88(1–2):141–5. doi: 10.1007/s00421-002-0692-3 12436282 10.1007/s00421-002-0692-3

[49] Sessler DI. Skin-temperature gradients are a validated measure of fingertip perfusion. Eur J Appl Physiol. 2003 5;89(3):401–2. 12736848 10.1007/s00421-003-0812-8

[50] Muzik O, Mangner TJ, Leonard WR, Kumar A, Janisse J, Granneman JG. ^15^O PET Measurement of Blood Flow and Oxygen Consumption in Cold-Activated Human Brown Fat. J Nucl Med. 2013 4 1;54(4):523–31. doi: 10.2967/jnumed.112.111336 23362317 10.2967/jnumed.112.111336 PMC3883579

[51] Nirengi S, Yoneshiro T, Sugie H, Saito M, Hamaoka T. Human brown adipose tissue assessed by simple, noninvasive near-Infrared time-resolved spectroscopy. Obesity. 2015;23(5):973–80. doi: 10.1002/oby.21012 25866030 10.1002/oby.21012

[52] Orava J, Nuutila P, Lidell MEE, Oikonen V, Noponen T, Viljanen T, et al Different Metabolic Responses of Human Brown Adipose Tissue to Activation by Cold and Insulin. Cell Metab. 2011;14(2):272–9. doi: 10.1016/j.cmet.2011.06.012 21803297 10.1016/j.cmet.2011.06.012

[53] Nedergaard J, Bengtsson T, Cannon B. Unexpected evidence for active brown adipose tissue in adult humans. Am J Physiol Endocrinol Metab. 2007;293:444–52. 10.1152/ajpendo.00691.2006 17473055

[54] Stocks J, Taylor N, Tipton M, Greenleaf J. Human physiological responses to acute and chronic cold exposure. Aviat Sp Environ Med. 2004;75(5):444–57. 15152898

[55] Matsumoto T, Miyawaki T, Ue H, Kanda T, Zenji C, Moritani T. Autonomic responsiveness to acute cold exposure in obese and non-obese young women. Int J Obes Relat Metab Disord. 1999 8 13;23(8):793–800. 10490779 10.1038/sj.ijo.0800928

[56] Liu W, Lian Z, Liu Y. Heart rate variability at different thermal comfort levels. Eur J Appl Physiol. 2008 6 20;103(3):361–6. doi: 10.1007/s00421-008-0718-6 18351379 10.1007/s00421-008-0718-6

[57] Blondin DP, Daoud A, Taylor T, Tingelstad HC, Bézaire V, Richard D, et al Four-week cold acclimation in adult humans shifts uncoupling thermogenesis from skeletal muscles to brown adipose tissue. J Physiol. 2017 3 15;595(6):2099–113. doi: 10.1113/JP273395 28025824 10.1113/JP273395 PMC5350439

[58] Blondin DP, Frisch F, Phoenix S, Guérin B, Turcotte ÉE, Haman F, et al Inhibition of Intracellular Triglyceride Lipolysis Suppresses Cold-Induced Brown Adipose Tissue Metabolism and Increases Shivering in Humans. Cell Metab. 2017 2;25(2):438–47. doi: 10.1016/j.cmet.2016.12.005 28089568 10.1016/j.cmet.2016.12.005

[59] Haman F, Péronnet F, Kenny GP, Massicotte D, Lavoie C, Scott C, et al Effect of cold exposure on fuel utilization in humans: plasma glucose, muscle glycogen, and lipids. J Appl Physiol. 2002;93(1):77–84. doi: 10.1152/japplphysiol.00773.2001 12070189 10.1152/japplphysiol.00773.2001

[60] Wu J, Boström P, Sparks LM, Ye L, Choi JH, Giang AH, et al Beige adipocytes are a distinct type of thermogenic fat cell in mouse and human. Cell. 2012;150(2):366–76. doi: 10.1016/j.cell.2012.05.016 22796012 10.1016/j.cell.2012.05.016 PMC3402601

[61] Himms-Hagen J, Melnyk A, Zingaretti MC, Ceresi E, Barbatelli G, Cinti S. Multilocular fat cells in WAT of CL-316243-treated rats derive directly from white adipocytes. Am J Physiol Cell Physiol. 2000;279(3):C670–81. doi: 10.1152/ajpcell.2000.279.3.C670 10942717 10.1152/ajpcell.2000.279.3.C670

[62] Lee YH, Petkova AP, Mottillo EP, Granneman JG. In vivo identification of bipotential adipocyte progenitors recruited by β3-adrenoceptor activation and high-fat feeding. Cell Metab. 2012;15(4):480–91. doi: 10.1016/j.cmet.2012.03.009 22482730 10.1016/j.cmet.2012.03.009 PMC3322390

[63] Lichtenbelt W van M, Kingma B, van der Lans A, Schellen L. Cold exposure—an approach to increasing energy expenditure in humans. Trends Endocrinol Metab. 2014 4;25(4):165–7. doi: 10.1016/j.tem.2014.01.001 24462079 10.1016/j.tem.2014.01.001

[64] Martinez-Tellez B, Sanchez-Delgado G, Acosta FM, Alcantara JMA, Boon MR, Rensen PCN, et al Differences between the most used equations in BAT-human studies to estimate parameters of skin temperature in young lean men. Sci Rep. 2017;7(1):1–12. 28874709 10.1038/s41598-017-10444-5 PMC5585347

