# Correction: Physiological responses to acute cold exposure in young lean men

**DOI:** 10.1371/journal.pone.0200865

**Published:** 2018-07-12

**Authors:** Francisco M. Acosta, Borja Martinez-Tellez, Guillermo Sanchez-Delgado, Juan M. A. Alcantara, Pedro Acosta-Manzano, Antonio J. Morales-Artacho, Jonatan R. Ruiz

There is an error in the citation. The fourth author’s name appears incorrectly. Please see the corrected citation here: Acosta FM, Martinez-Tellez B, Sanchez-Delgado G, Alcantara JMA, Acosta-Manzano P, Morales-Artacho AJ, et al. (2018) Physiological responses to acute cold exposure in young lean men. PLoS ONE 13(5): e0196543. https://doi.org/10.1371/journal.pone.0196543
