# Supplementary material for: Physiological responses to acute cold exposure in young lean men
Source: PLoS One. 2018 May 7;13(5):e0196543. doi: 10.1371/journal.pone.0196543 (PMC5937792; doi:10.1371/journal.pone.0196543)
Supplement: S1 Fig — 26 different positions can be distinguished. Panel (A): distribution of the iButtons over the whole body, Panel (B): distribution of the iButtons on the right clavicular sites. (PDF) [file pone.0196543.s001.pdf]

SUPPORTING INFORMATION

S1 Fig

(A) **(B)**

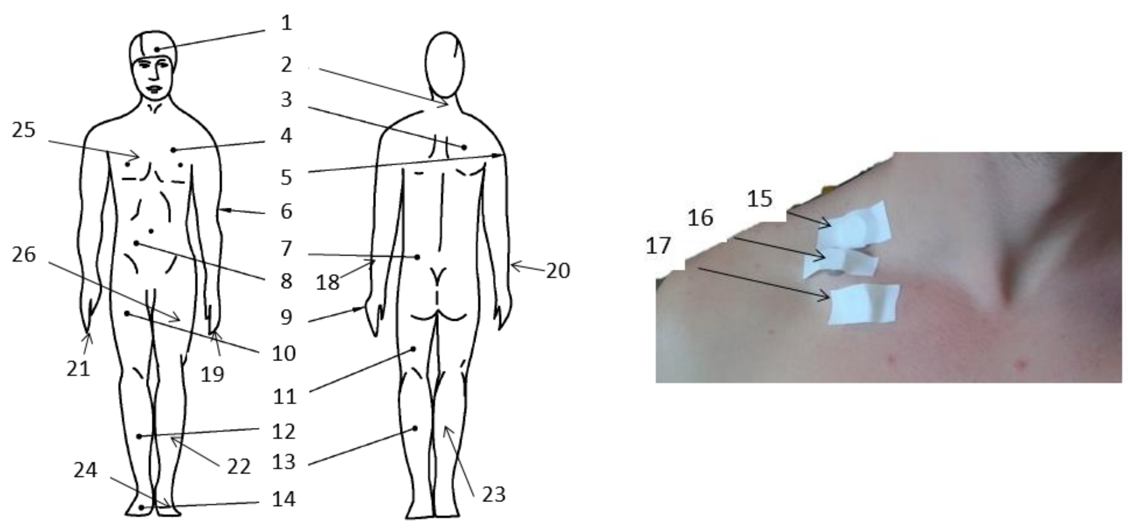

S1 Fig. Body anatomical points where iButtons attached to skin. 26 different positions can be distinguished. Panel (**A**): distribution of the iButtons over whole body, Panel (**B**): distribution of the iButtons on the right clavicular sites.
