# Supplementary material for: Physiological responses to acute cold exposure in young lean men
Source: PLoS One. 2018 May 7;13(5):e0196543. doi: 10.1371/journal.pone.0196543 (PMC5937792; doi:10.1371/journal.pone.0196543)
Supplement: S1 Table — Table adapted from Martinez-Tellez et al. [64]. (PDF) [file pone.0196543.s002.pdf]

SUPPORTING INFORMATION

S1 Table. Equations used to measure skin temperature

Outcome	Reference	iButtons (n)	Anatomical positions. S1 Fig	Equations
**Mean skin temperature**	14 ISO 9886-2004 (20) *(14-ISO)*	14	From 1 to 14	(Forehead*0.07)+(Neck*0.07)+(Right Scapula*0.07)+(Left Chest*0.07)+(Right Deltoides*0.07)+(Left Elbow*0.07)+(Right Abdomen*0.07)+(Left Hand*0.07)+(Left Lumbar *0.07)+(Right Thigh*0.07)+(Left Harmstring*0.07)+(Right Shin bone*0.07)+(Left Gastrocnemius*0.07)+(Right Instep*0.07)
**Proximal skin temperature**	Boon et al. (21)*(Boon)*	3	10,16,8	(Right Thigh*0.383)+(Right Clavicular*0.293)+(Right Abdomen*0.324)
**Distal skin temperature**	Kräuchi et al. (22) *(Krauchi)*	2	9, 14	(Left Hand+Right Instep)/2
**Body temperature gradient**	Boon et al. (21)*(Boon)*	5	9,14,10,16,8	[(Left Hand+Right Instep)/2]- [(Right Thigh*0.383)+(Right Clavicular*0.293)+(Right Abdomen*0.324)]
**Supraclavicular temperature gradient**	Lee et al. (24)*(Lee S-RC)*	2	15, 25	(Right Supraclavicular(S)- Right Chest (RC))
**Peripheral temperature Gradient**	Sessler et al. (23) Right arm	2	20, 21	(Right Forearm-Right Top of forefinger)

Table adapted from Martinez-Tellez et al. (64).
