# Supplementary material for: Physiological responses to acute cold exposure in young lean men
Source: PLoS One. 2018 May 7;13(5):e0196543. doi: 10.1371/journal.pone.0196543 (PMC5937792; doi:10.1371/journal.pone.0196543)
Supplement: S2 Table — Values are mean ± standard deviation (n = 7). Repetead measures analysis of variance was performed, using Bonferroni post-hoc tests for pairwise comparisons. No significant differences were observed across periods (P>0.05). Mean RR: mean length of all RR intervals, pNN50: percentage of consecutive normal RR intervals differing more than 50 ms, RMSSD: square root of the mean squared sum of the differences of successive NN intervals, SDNN: standard deviation of all RR legnth intervals, ST: shivering thereshold period, WP: warm period, 31% and 64%: percentage of the individual’s time exposed to cold until shivering occurred. (PDF) [file pone.0196543.s003.pdf]

SUPPORTING INFORMATION

S2 Table. Time domain parameters of heart rate variability rate across study periods.

	WP			31%			64%			ST		
**Mean RR (ms)**	1064.9	±	1.8	1086.0	±	105.2	1069.9	±	112.1	1025.4	±	121.6
**SDNN**	70.1	±	6.2	76.4	±	22.5	70.6	±	20.9	81.6	±	21.3
**RMSSD (ms)**	78.0	±	1.2	91.9	±	31.1	88.6	±	34.3	88.1	±	37.2
**pNN50 (%)**	50.6	±	8.6	57.1	±	5.8	54.6	±	15.2	46.6	±	17.5

Values are mean ± standard deviation (n = 7). Repetead measures analysis of variance was performed, using Bonferroni correction for pairwise comparisons. No significant differences were observed across periods (P>0.05). Mean RR: mean length of all RR intervals, pNN50: percentage of consecutive normal RR intervals differing more than 50 ms, RMSSD: square root of the mean squared sum of the differences of successive NN intervals, SDNN: standard deviation of all RR legnth intervals, ST: shivering thereshold period, WP: warm period, 31% and 64%: percentage of the individual’s time exposed to cold until shivering occurred.
